# The Journey of DDR1 and DDR2 Kinase Inhibitors as Rising Stars in the Fight Against Cancer

**DOI:** 10.3390/ijms22126535

**Published:** 2021-06-18

**Authors:** Ahmed Elkamhawy, Qili Lu, Hossam Nada, Jiyu Woo, Guofeng Quan, Kyeong Lee

**Affiliations:** 1 College of Pharmacy, Dongguk University-Seoul, Goyang 10326, Korea or a_elkamhawy@mans.edu.eg (A.E.); luqililily@dgu.ac.kr (Q.L.); hossam_hammouda@dgu.ac.kr (H.N.); wju2757@dgu.ac.kr (J.W.); quan0922@dgu.ac.kr (G.Q.); 2 Department of Pharmaceutical Organic Chemistry, Faculty of Pharmacy, Mansoura University, Mansoura 35516, Egypt

**Keywords:** discoidin domain receptor (DDR), cancer, kinase inhibitors, structure-activity relationship (SAR), DDR1 and DDR2

## Abstract

Discoidin domain receptor (DDR) is a collagen-activated receptor tyrosine kinase that plays critical roles in regulating essential cellular processes such as morphogenesis, differentiation, proliferation, adhesion, migration, invasion, and matrix remodeling. As a result, DDR dysregulation has been attributed to a variety of human cancer disorders, for instance, non-small-cell lung carcinoma (NSCLC), ovarian cancer, glioblastoma, and breast cancer, in addition to some inflammatory and neurodegenerative disorders. Since the target identification in the early 1990s to date, a lot of efforts have been devoted to the development of DDR inhibitors. From a medicinal chemistry perspective, we attempted to reveal the progress in the development of the most promising DDR1 and DDR2 small molecule inhibitors covering their design approaches, structure-activity relationship (SAR), biological activity, and selectivity.

## 1. Introduction

Discoidin domain receptor (DDR), discovered in the early 1990s, belongs to a family of the transmembrane receptor tyrosine kinases (RTKs) which acts as a hub for signal transduction initiation. A discoidin motif (DS) which encompasses the collagen-binding site is a specific structural feature that distinguishes the human DDRs (DDR1 (CD167a) and DDR2 (CD167b)) from other RTKs. While DDR1 has five isoforms (DDR1a, DDR1b, DDR1c, DDR1d, and DDR1e) different in the extent of glycosylation, protein interactions, expression patterns, phosphorylation, as well as functions, DDR2 has only a single isoform to date. DDR1a, b, and c are found to be kinase-active, while DDR1d and e are kinase domain-deficient receptors with unknown function. It is well-established that ligands of typical RTKs are peptide-like growth factors, on the other hand, DDR activation is controlled by numerous types of triple-helical collagens. DDR1 is mainly expressed in epithelial cells of different tissues while DDR2 is found in mesenchymal cells including fibroblasts, myofibroblasts, smooth muscle cells, and chondrocytes. DDR plays a key role in the production and degradation processes of collagen and the essential cellular processes such as proliferation, differentiation, adhesion, in addition to matrix remodeling [1,2,3].

Collective evidence suggests that dysregulation of DDR is attributed to different human disorders, such as cancer, fibrosis, atherosclerosis, neurodegeneration, and other inflammatory disorders. Accordingly, DDR has been considered as novel potential molecular target, mainly for drug development of cancer. Many DDR inhibitors have been disclosed highlighting the promising potential of DDR inhibition as a novel therapeutic strategy [4,5]. From a medicinal chemistry perspective, this review offers an updated overview on the development journey of the most promising DDR small molecule inhibitors including design, structure-activity relationship (SAR), biological activity, and selectivity.

## 2. Structure and Activation of DDR Kinase

**Figure 1:**
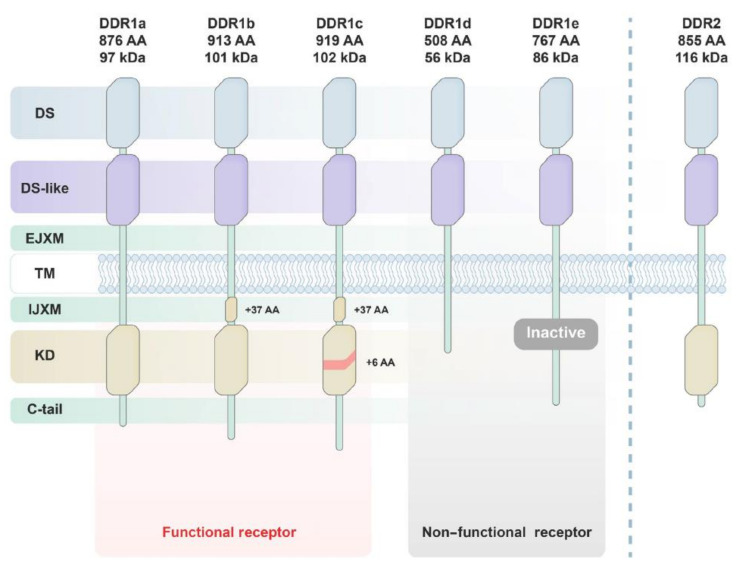
Structures and subtypes of DDR1 and DDR2. Reprinted from with permission Ref. [13].

Similar to the classic RTKs, which share high structural features, the molecular structure of DDR consists of three main domains: an extracellular binding one, a transmembrane domain (TM), and an intracellular domain (Figure 1). In the extracellular part of DDR, there are two main components: a unique DS motif and a DS-like domain responsible for the collagen binding. The TM domain consists of an extracellular juxtamembrane (JM) region possessing phosphorylatable tyrosine amino acids (the docking site for DDR binding proteins), and a TM helix which arbitrates collagen independent receptor dimerization. The intracellular domain contains an intracellular JM region and a catalytic tyrosine kinase domain which controls the intrinsic enzymatic activity [6]. The kinase domains of DDR1 and DDR2 were discovered to have a high degree of sequence and morphological homology with those of Abl and c-Kit kinases [7]. Glycine-rich loop, A-loop, DFG motif, and C-helix are among the loops or motifs found in the DDR1 kinase domain, which are also found in other RTKs [8]. However, DDR1 and DDR2 are activated by different forms of collagen rather than soluble growth factors, unlike other RTKs [9]. Collagen binding activates the tyrosine kinase Src to phosphorylate tyrosines in the DDR activation loop. Accordingly, the activation of kinase domain in DDR autophosphorylates numerous additional tyrosines in the JM region, which subsequently leads to the recruitment of downstream adaptor proteins to regulate cell behavior. In comparison to ligand-induced dimerization in other RTKs, intrinsic DDR dimerization occurs without recognizing the ligand [10]. Furthermore, while typical RTK activation is triggered in just a few seconds after binding to a ligand, DDR phosphorylation is noticeably slow; several hours are needed to get full activation [9,11]. An additional insulin-like growth factor (IIGF) system-based activation model of DDR was recently discovered. In a collagen-independent manner, insulin, IGF1, and IGF2 were found to upregulate DDR1 expression and phosphorylation [3,12].

## 3. Biological Role of DDR

Both DDR1 and DDR2 are essential regulatory factors for organ development and physiological function [9,14,15,16,17,18]. In addition to their important roles in cell proliferation and differentiation, DDRs were also found to have rules in cell migration, invasion, and adhesion [10,19,20,21]. DDR1 has an essential role in the biogenesis of multiple organs, for example, DDR1-knockout mice were found to be shorter than their littermates and to have a lactational defect in pregnant females. Multiple reproductive disorders, including infertility due to abnormal embryo implantation and abnormal mammary gland growth, were also discovered in DDR1-null mice [22,23]. In addition to extreme auditory function loss and progressive morphological changes, they displayed abnormalities in kidney and inner ear architecture [24]. DDR1 deficiency impairs adhesion and migration abilities [25]; it was reported that DDR1a is an important factor for the promotion of leukocyte migration in three-dimensional collagen lattices [20]. DDR1 mediated activated T cells were also found to bind to collagen, which enhanced T cell migration [26].

DDR2 was reported to be involved in skeletetogenesis since it was found to be important for chondrocyte proliferation [27,28]; In DDR2-null mice, skeletal disorders such as the shortening of long bones and abnormal growth of flat bones have recently been reported [29]. Another study by Kano et al., showed the critical role of DDR2 signaling in the maintenance of male spermatogenesis [30]. Skin wound-healing disorders were also observed in DDR2-knockout mice, which were primarily caused by decreased skin fibroblast proliferation and abnormal extracellular matrix remodeling [21]. Furthermore, a link has been found between DDR2 deletion or mis-sense mutation and autosomal recessive growth disorders such as Smallie (Slie) and human spondylo-meta-epiphyseal dysplasia, which is characterized by short limbs and irregular calcifications (SMED-SL) [31,32,33,34,35]. Overexpression of DDR2 promotes the proliferation and invasion of hepatic stellate cells mediated by matrix metalloproteinase-2 (MMP-2) [36]. It was confirmed that DDR2 is necessary for normal fibroblast spreading and migration, regardless of the presence of adhesion ligands or collagen activation [37]. Studies also suggested that DDR2 function is essential for the membrane dynamics that control the mechanical attachment of fibroblasts to the 3D collagen matrices [38]. DDR2 reduction was also found to increase the population of CD8+ T cells as well as the sensitivity to anti–programmed cell death protein 1 (PD-1) therapy [39].

## 4. Role of DDR in Cancer

DDR1 and DDR2 dysregulation has been linked to multiple forms of cancer. Many studies have shown that elevated DDR expression levels and/or mutations can be found in a number of cancer cell lines as well as primary tumor tissues including lung [40,41,42,43,44,45,46,47], pancreas [48], prostate [49], breast [50,51], brain [52,53], ovary [54,55], liver [56], and others [57,58,59,60,61,62,63]. DDR1 was found to be a prognostic marker for non-small-cell lung carcinoma (NSCLC) patients. A clinicopathological parameter analysis in NSCLC patients presented a significant connection between DDR1 overexpression and lymph node metastasis [64]. A recent study by Reger de Moura et al., demonstrated that siRNA-mediated downregulation of DDR1 suppressed melanoma cell malignancy, migration, invasion, and survival [65]. DDR1 protein was also found to be expressed in 63% of serous ovarian cancer tissue, but not in normal ovarian surface epithelium [55]. Ma et al., also found an important role of DDR1 in glioblastoma cell invasion and epithelial-mesenchymal transition (EMT) [66]. According to a recent study by Hur et al., DDR1 expression was found in 50.5% of gastric cancer tissues [67]. Furthermore, a combination of DDR1 and Notch signaling inhibitors has been shown to be an effective treatment option for patients with K-Ras-mutant lung adenocarcinoma [68]. DDR1 was found to control triple-negative breast cancer growth by modulating tumor-infiltrating CD4+ and CD8+ T cells [69]. There is also strong evidence indicating that DDR2 could be a potential biomarker and a molecular target for a variety of cancer disorders. For instance, DDR2 overexpression was reported to contribute to NSCLC [44,46,64], thyroid carcinoma [70,71], Hodgkin’s lymphoma [72,73], nasopharyngeal carcinomas [57,71], prostate cancer, as well as to head and neck squamous cell carcinoma [74,75]. DDR2 contributes to breast cancer metastasis by stabilizing the SNAIL1 protein, according to mode of action studies reported by Zhang et al. [76]. DDR2 has also been shown to be a favorable independent predictor of recurrence and outcome in primary breast cancers. [77,78]. In addition to the essential roles of the wild type of DDR in cancer pathology and prognosis, various mutations of DDR1 and/or DDR2 have also been reported in numerous types of cancer cells, for instance, G1486T(DDR1) and A496S(DDR1) in lung cancer [44], N502S(DDR1), A533S(DDR1), and A803V(DDR1) in acute myeloid leukemia (AML) [79,80], and S768R(DDR2) in squamous cell carcinoma [81].

DDRs also play a role in cancer growth by controlling how tumor cells interact with their surrounding collagen matrix [5]. This role of DDRs becomes more prominent when considering their role as extracellular matrix receptors. The extracellular matrix (ECM) confers structural properties to tissues around the tumor, as well as regulating cell proliferation, survival, migration, and invasion [82]. The physiological interactions between tumor cells and their immediate microenvironment, represented by the extracellular matrix, are disrupted in metastatic cancers. As a key component of the tumor extracellular matrix, type I collagen shows high density and distorted architecture in malignant cancer, linking it to tumor formation and metastasis [83]. Therefore, the discovery of DDRs as collagen receptors represents a new target in the regulation of tumor progression [84,85,86,87,88,89].

## 5. Role of DDR in Inflammation and Neurodegenerative Disorders

Aside from the main role of DDR1 and DDR2 in human cancer, they are also involved in other disorders such as inflammation, tissue fibrosis and atherosclerosis, and neurodegenerative diseases [5,90,91,92]. A study by Matsuyama et al., reported that DDR1 not only stimulated inflammatory factor secretion, but it also enhanced the effects of other stimuli including proinflammatory cytokines or bacterial products [93]. Another study found that DDR1 null mice’s renal cortical slices had a blunted chemokine response to lipopolysaccharide (LPS), as well as significant defense against LPS-induced mortality, implying that DDR1 is an essential mediator of inflammation [94]. Gross et al., demonstrated that DDR1 was expressed in glomerular epithelial cells (podocytes) where its loss or downregulation reduced the total amount of transforming growth factor-β1 (TGFβ) and connective tissue growth factor (CTGF) within the kidney. Furthermore, in inherited type IV collagen disease, loss of DDR1 expression in the kidney delayed renal fibrosis and inflammation [95]. DDR1 deletion was also found to be effective in reducing bleomycin-induced lung inflammation and pulmonary fibrosis [96]. In various renal disease models, such as hypertensive nephropathy and glomerular nephritis, inhibition of DDR expression was found to prevent the production of renal inflammation and fibrosis, as well as maintain renal structure. Reduced DDR2 has also been shown to slow the progression of osteoarthritis in knee joints [97]. These findings indicated that DDR inhibition may be a promising new treatment option for inflammatory diseases.

Although the function of DDRs in neurodegeneration is unclear, they have been found to be upregulated in Alzheimer’s and Parkinson’s diseases (AD and PD) [98]. A study by Zhu et al., reported DDR1 to be found in the central nervous system (CNS) and has been linked to the regulation of microglial activity and MMPs, as well as the degradation of the blood–brain barrier (BBB) [99]. Using multiple models of neurodegeneration and DDR1 knockout mice, some potent and preferential DDR1 inhibitors were able to reduce neurotoxic protein levels in vitro and in vivo [4]. In a mouse model confronted with α-synuclein, partial or full deletion or inhibition of DDR1 improved autophagy and decreased inflammation and neurotoxic proteins. Some potent DDR1 inhibitors such as nilotinib and LCB-03-0110 were also found to lower amyloid-β (Aβ), hyperphosphorylated tau (p-tau), and α-synuclein levels in the CNS while increasing dopamine levels [100,101,102,103,104,105,106]. Thus, DDR1 inhibition, which succeeded to decrease neurotoxic proteins and inflammation was demonstrated as a potential therapeutic approach in neurodegeneration.

## 6. Small Molecule DDR Kinase Inhibitors

**Figure 2:**
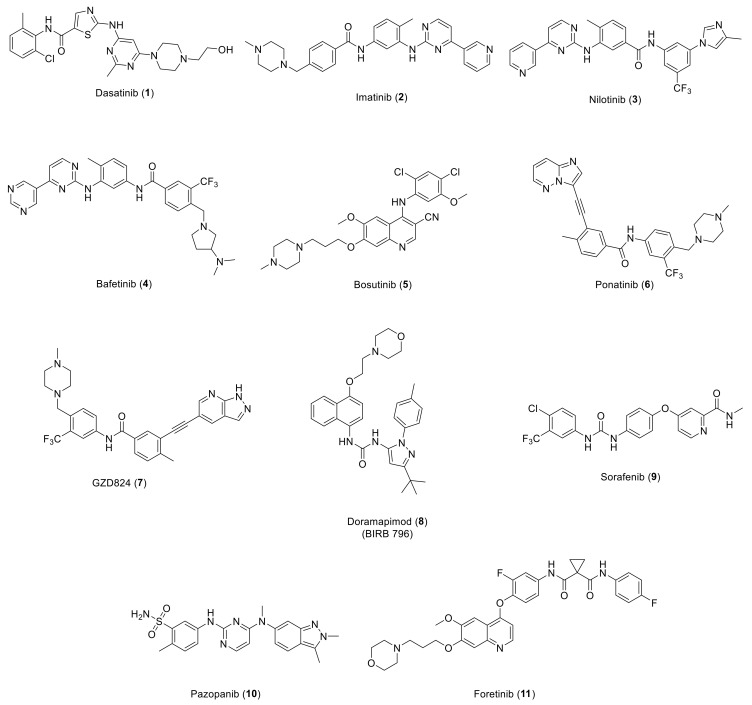
Chemical structures of compounds **1**–**11**.

Overexpression of various types of RTKs is found in different types of cancer, which encouraged medicinal chemists worldwide to develop numerous RTKs inhibitors [107,108,109,110,111,112,113,114,115,116,117,118,119,120]. Among these efforts, several DDR kinase inhibitors have been discovered so far, some of them have been demonstrated to possess a promising therapeutic potential. With the aim to reveal the developmental journey of small molecule DDR inhibitors, we selected the most promising potent and/or selective small molecules to present. However, most of the reported DDR1/2 inhibitors have a broad inhibition over several kinases with limited potency. Since the majority of kinase inhibitors bind within the ATP binding region, unintended inhibitor off-target binding can occur due to high structural homology across kinase ATP binding pockets. As a result, a number of Bcr-Abl inhibitors (Figure 2) including dasatinib (**1**), imatinib (**2**), and nilotinib (**3**) were found to inhibit DDR1b kinase and DDR2 with IC_50_ values of 0.5, 337, 43 nM, and 1.4, 675, 55 nM, respectively [7]. Further research revealed that these three compounds inhibited collagen-induced autophosphorylation of DDR1b and suppressed monocyte chemoattractant protein-1 (MCP-1) release in monocytic cells in a DDR1 dependent manner [7]. A comprehensive drug-protein interaction profiles for compounds **1**–**3** confirmed their strong DDR inhibitory effects via a global chemical proteomics approach [121]. Another study on lung cancer cells harboring “gain-of-function” mutations of DDR2 reported compound **1** to demonstrate a highly promising therapeutic efficacy [46]. Moreover, a number of squamous cell carcinoma (SQCC) patients with S768R mutation of DDR2 had substantial tumor shrinkage following treatment with compound **1** [81,122,123,124]. Other type II Bcr-Abl inhibitors, for instance, bafetinib (**4**), bosutinib (**5**), ponatinib (**6**), and GZD824 (**7**), have also been shown to potently inhibit DDR [125,126,127,128]. The X-ray structures of DDR1 cocrystals containing ligands **2** and **6** show that the compounds bind to inactive DDR1 in a type II binding mode close to that of Bcr-Abl kinase [8]. Numerous other kinase inhibitors were found to show non-selective inhibitory activity over DDR (Figure 2) [129]; doramapimod (BIRB 796, **8**), a potent p38 MAPK inhibitor, was reported to bind to DDR1 and DDR2 in highly potent nanomolar range (*K*_d_ = 1.9 and 33 nM, respectively). Sorafenib (**9**), a B-Raf/ VEGFR dual inhibitor approved for advanced hepatocellular carcinoma, was found to bind to DDR1 and DDR2 with *K*_d_ values of 1.5 and 6.6 nM, respectively. It was also reported that pazopanib (VEGFR inhibitor, **10**) was able to bind to DDR2 with *K*_d_ value of 57 nM. Foretinib, another c-Met/VEGFR-2 dual inhibitor (**11**), was shown to potently bind to DDR1with a *K*_d_ value of 0.2 nM.

**Figure 3:**
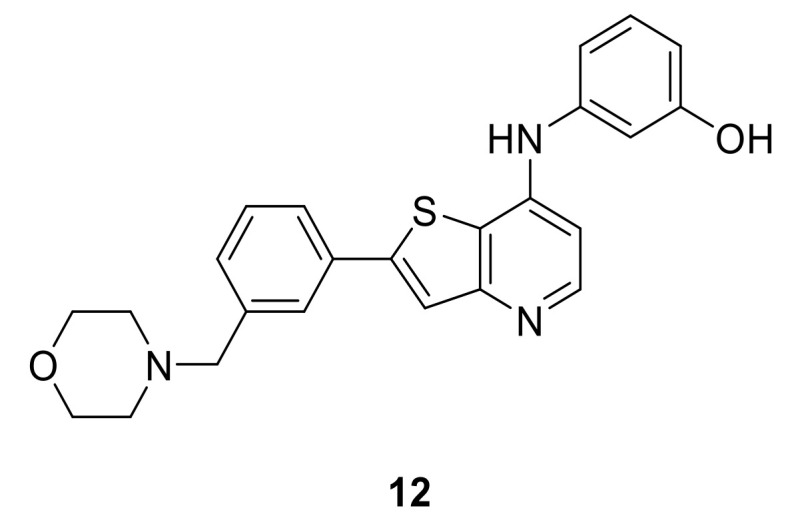
Chemical structure of compound **12**.

LCB 03-0110 (**12**, Figure 3) is a thienopyridine derivative that inhibits many tyrosine kinases, including the c-Src family, Btk, VEGFR-2, and DDR kinases, all of which have been implicated in fibroblast and macrophage activation. DDR2 active form was inhibited by LCB 03-0110 at IC_50_ value of 6 nM, while the non-activated form was inhibited at IC_50_ value of 145 nM, indicating that LCB 03-0110 is more inhibitory to the active form. The kinetics assay of LCB 03-0110 against the active DDR2 tyrosine kinase revealed that inhibition is ATP-competitive. In a cell-based assay, LCB 03-0110 suppressed DDR1 and DDR2 induced autophosphorylation in HEK293 cells designed to overexpress either HEK293-DDR1b or HEK293-DDR2 with IC_50_ values of 164 and 171 nM, respectively. Moreover, LCB 03-0110 inhibited more than 90% of 20 tyrosine kinases at 10 µM in a kinase panel assay against 60 kinases, suggesting LCB 03-0110 as a multiple tyrosine kinase inhibitor. In addition, LCB 03-0110 was reported to suppress the proliferation and migration of primary dermal fibroblasts, as well as inhibiting cell migration, and nitric oxide, iNOS, COX-2, and TNF-α synthesis, suggesting its role as a novel anti-fibro-inflammatory agent via suppressing fibro-inflammation by concurrently targeting activated fibroblasts and macrophages [130].

**Figure 4:**
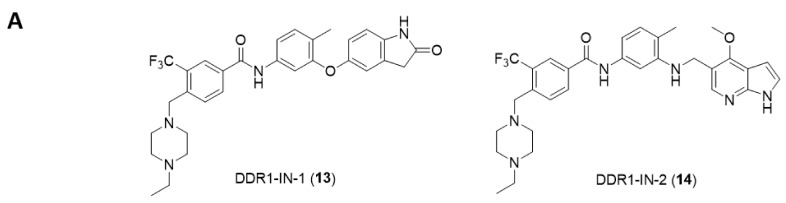

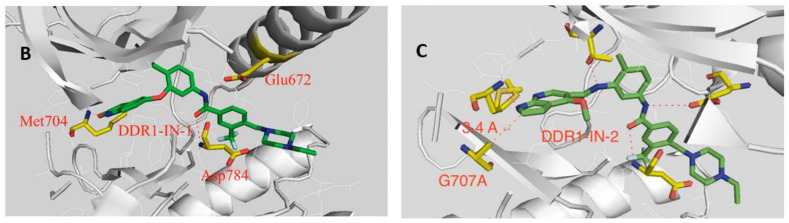
(**A**) Chemical structures of compounds **13** and **14**; (**B**) X-ray co-crystal structure of compound **13** with DDR1 kinase. Reprinted with permission from Ref. [133]; (**C**) Compound **14** docking model into the DDR1 G707A mutation. Reprinted with permission from Ref. [132].

Gray et al., from Harvard Medical School designed a general pharmacophore model for type II kinase inhibitors to develop a library of potential kinase inhibitors [131] which led to the discovery of the potent and selective DDR1 inhibitor DDR1-IN-1 (**13**, Figure 4A) [132,133]. It was found that DDR1-IN-1 was able to bind to DDR1 in the DFG-out conformation and inhibited DDR1 autophosphorylation in cells at submicromolar concentrations with promising selectivity as assessed over a panel of 451 kinases. While DDR1-IN-1 showed IC_50_ values of 105 nM against DDR1 and 413 nM against DDR2, its analog DDR1-IN-2 (**14**, Figure 4A) demonstrated IC_50_ values of 47 and 145 nM against DDR1 and DDR2, respectively. Despite the high potency of DDR1-IN-2 over DDR1, it also potently inhibits a number of additional kinases. The corrected X-ray cocrystal structure of DDR1-IN-1 with DDR1 kinase (PDB: 4CKR) confirmed the presumed type II binding mode (Figure 4B) where electron density analysis showed that the indolin-2-one group is flipped, forming only a single hydrogen bond to Met704. The DDR1 crystal structure with a modeled G707A mutation indicated the presence of a potential clash between the side-chain methyl group of alanine and the azaindole ring of DDR1-IN-2 (Figure 4C) and predicted that Thr701 (gatekeeper) has the potential to form a hydrogen bond with nitrogen between the “Head” and “Linker” region of DDR1-IN-2, clarifying its ability to more potently inhibit DDR1 relative to DDR1-IN-1. In a mechanistic study comparing the inhibition of DDR1 by different kinase inhibitors by Canning et al., imatinib (**2**) and ponatinib (**6**) were able to bind potently to both the DDR and ABL kinases, while DDR1-IN-1 appears to fail to satisfy the hydrophobic interactions of the ABL P-loop, implying a structural basis for its DDR1 selectivity [8].

**Figure 5:**
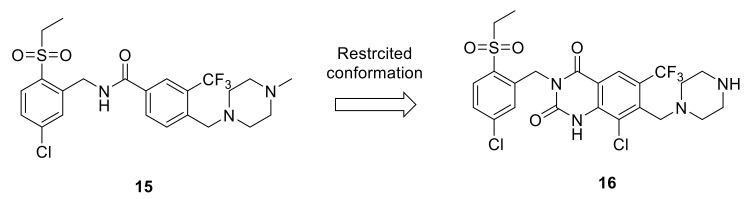
Restricted conformation strategy and chemical structures of compounds **15** and **16**.

Takeshi et al., identified some benzamide and quinazolindione analogs as novel DDR1 inhibitors [134,135] (Figure 5). Compound **15** was found to exhibit a high inhibitory activity over DDR1 kinase (IC_50_ = 0.097 µM) and suppressed the proliferation in U2OS cells with DDR1 overexpression with an IC_50_ value of 0.44 µM. The conformationally restricted quinazolindione analog **16** was also able to inhibit the kinase activity of DDR1 with a more potent IC_50_ value (0.043 μM). Interestingly, both compounds exhibited promising in vivo anticancer activity.

**Figure 6:**
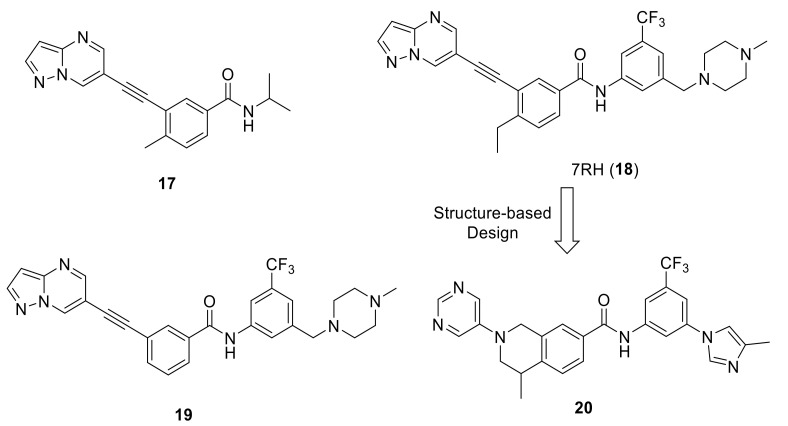
Chemical structures of DDR1inhibitors **17**, **18** (7RH), **19**, and **20**.

In 2013, Gao et al., reported a new series of 3-(2-(pyrazolo[1,5-a]pyrimidin-6-yl)ethynyl)benzamides as selective DDR1 inhibitors [136]. Compound **17** (Figure 6) was selected as the lead molecule for extensive structural optimization since it demonstrated a promising selective DDR1 inhibition (IC_50_ = 39.6 nM). All the synthesized derivatives were tested for their inhibitory activity against four kinases (DDR1, DDR2, Bcl-Abl and c-Kit). Among all, only compounds **18** (7RH) and **19** (Figure 6) showed potent selective activity against DDR1 with IC_50_ values of 6.8 and 7.0 nM, respectively. Compound 7RH was able to interact with the ATP-binding site of DDR1 with a *K*_d_ value of 0.6 nM. It also showed high selectivity for DDR1 upon its screening over a large kinase panel of 456 kinases. Both compounds (**18** and **19**) demonstrated low IC_50_ values over NCI-H23 NSCLC cell line expressing high level of DDR1 and promising oral bioavailability (67.4% and 56.2%, respectively). Three years later, Lu et al., reported the antitumor activity of 7RH alone or in combination with dasatinib in nasopharyngeal carcinoma (NPC) [137]. 7RH demonstrated cytotoxicity in CNE2, HONE1, CNE1 and SUNE1 (NPC cell lines) with IC_50_ values of 1.97, 3.71, 2.06, and 3.95 µM, respectively. It also reduced protein expression levels, induced cell cycle arrest and apoptosis of CNE2 cells, and inhibited NPC cell adhesion at different concentrations. 7RH and dasatinib were found to exhibit a synergistic inhibitory effect over human NPC cell proliferation. 7RH, dasatinib and 7RH + dasatinib groups showed 27, 28 and 33% in vivo tumor growth inhibitory rates, respectively.

Using compound 7RH (**18**) as a lead compound, a structure-based design of new tetrahydroisoquinoline-7-carboxamide derivatives as selective DDR1 inhibitors was reported by Wang et al. [138]. Among 11 target derivatives assessed for their inhibitory activity against two kinases (DDR1 and Abl1), compound **20** (Figure 6) was the most promising compound over DDR1 (IC_50_ = 9.4 nM and *K_d_* = 4.7 nM). Moreover, remarkable pharmacokinetic (PK) properties were demonstrated with an oral bioavailability of 66.8%. To examine the target specificity of compound **20**, a kinase selectivity profiling analysis was performed at 1.0 M against a panel of 468 kinases (including 403 nonmutated kinases). The outcomes demonstrated an outstanding target selectivity. In addition, it exhibited an inhibitory effect of DDR1-mediated signaling in a concentration dependent manner in PHLF (Primary Human Lung Fibroblast) and showed a blocking effect in BLM-induced lung fibrosis. The high potency along with the unique target specificity of compound **20** presents a great opportunity for biological target investigations and new drug discovery programs.

**Figure 7:**
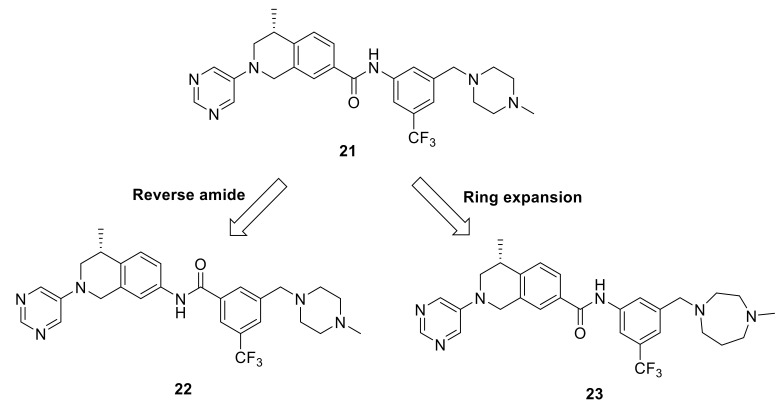
Drug design approaches and chemical structures of compounds **21**–**23**.

**Figure 8:**
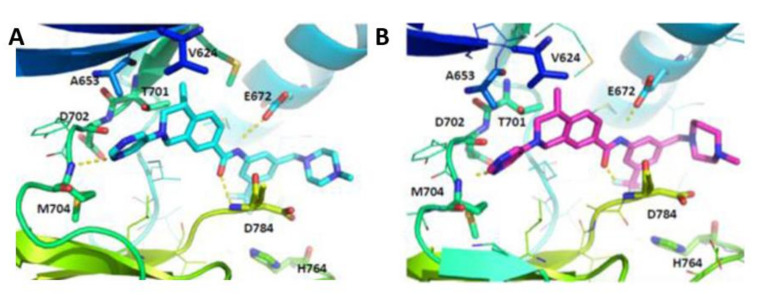
Docking models of compounds **21** (**A**) and **23** (**B**) into DDR1 binding pocket. Reprinted from Ref. [139].

As a continuation of their research, Wang et al., optimized the tetrahydroisoquinoline-7-carboxamide-based scaffold [139]. Compound **21** [138] (Figure 7) was chosen as a lead molecule for optimization followed by an extensive SAR investigation. It was found that the trifluoromethylphenyl group in **21** was able to bind to the hydrophobic pocket formed by the DFG-out conformation of DDR1 (Figure 8A). The replacement of the trifluoromethylphenyl group with cyclopropyl or cyclohexyl substitutes, or its removal resulted in compounds with an obvious loss of potency, indicating that a lipophilic substitution at this position is critical to retain the potent DDR1 inhibition. The modeling studies also revealed that the amide linker in **21** bound deeply with Glu672 in the C-helix and Asp784 in the DFG motif by forming two pairs of hydrogen bonds. With the aim to evaluate of the contribution of these hydrogen bonds to DDR1 inhibition, compound **22** (Figure 7) with a reverse amide was made and evaluated. It turned out that compound **22** displayed a three-fold less potency than that of **21**. The reversed amide linker forced the trifluoromethylphenyl group to moderately rotate away from the C-helix, leading to the longer distances between amide moiety and the corresponding Glu672 and Asp784 residues with values of 2.5 and 2.3 Å. Accordingly, the hydrogen bond between **22** with the corresponding amino acids might be weaker than that of **21**, which explains the potency loss of compound **22**.

The most active compound in this series (**23**, Figure 7) with 1-methylhomopiperazinemethyl substituent was discovered to tightly bind to DDR1 protein (Figure 8B) with a *K*_d_ value of 2.2 nM and IC_50_ value of 6.6 nM. Compound **23** demonstrated a promising target specificity and suppressed LPS-induced interleukin-6 (IL-6) and TNF-α release in a dose-dependent manner suggesting it as a promising in vivo anti- inflammatory agent. Furthermore, it also inhibited the LPS-induced increase in total cell number and total protein concentration in bronchial alveolar lavage fluid (BALF). Accordingly, compound **23** was found not only to be a potent DDR1 inhibitor, but also a potential lead candidate for anti-inflammatory drug discovery.

**Figure 9:**
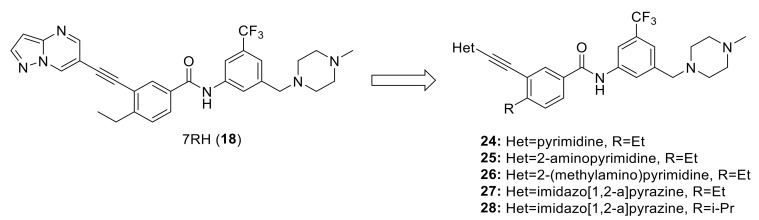
Chemical structures of compound **24**–**28** designed based on 7RH (**18**) as a lead molecule.

Wang et al. also recently described a new series of dual DDR1/2 inhibitors (some of which are illustrated in Figure 9, **24**–**28**) based on their starting lead 7RH (**18**). A series of 18 compounds were obtained as selective dual DDR1/2 inhibitors [140]. Among them, compound **28** turned out to be one of the most specific DDR1/2 dual inhibitors to-date, with IC_50_ values of 9.4 and 20.4 nM and *K_d_* values of 7.9 and 8.0 nM, respectively. It also exhibited a dose-dependently inhibitory effect on LPS-induced IL-6 release in mouse primary peritoneal macrophages (MPMs) and attenuated the lung inflammation in LPS-treated mice. It is worthy to mention that this study was the first in vivo investigation to find selective dual inhibitors (DDR1 and DDR2) as novel anti-inflammatory candidates.

**Figure 10:**
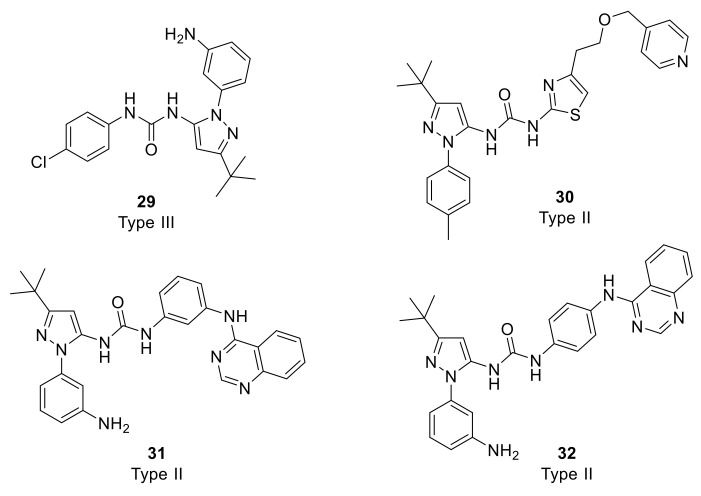
Chemical structures of type II and III DFG-out binders for DDR2 (**29**–**32**).

Richters et al. designed a fluorescence-based direct binding assay for the identification of DDR2 type II and III DFG-out binders [141]. While a total number of 852 compounds from internal library were screened, seven candidates including compound **29** (Figure 10) belonging to type III-like inhibitors were found to bind tightly to DDR2 with *K_d_* values of 0.015–0.702 μM. However, they exhibited moderate IC_50_ values of 0.183–4.70 μM. On the other hand, type II-like analogs (**30**, **31**, and **32**, Figure 10) showed excellent binding effects with *K_d_* values of 0.020–0.063 μM and potent IC_50_ values of 0.008–0.029 μM. Type II-like molecules also exhibited inhibitory effect on DDR1 with IC_50_ values 0.039–0235 μM. In addition, the inhibitory effect of compound **32** against the gatekeeper mutations was found to be conserved, for instance, it exhibited an IC_50_ value of 0.002 μM against DDR2 (T654M). Accordingly, compound **32** was suggested to offer a future approach towards the development of novel inhibitors that can overcome the potential resistant variants of DDR.

**Figure 11:**
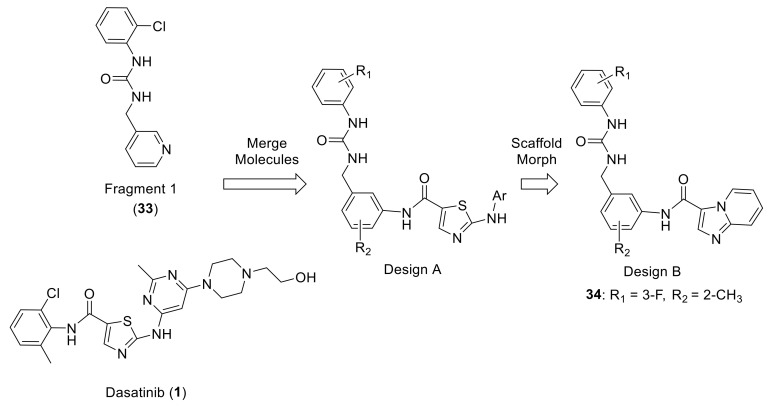
Schematic of the design process leading to discovery of compound **34**. Reprinted with permission from Ref. [142].

**Figure 12:**
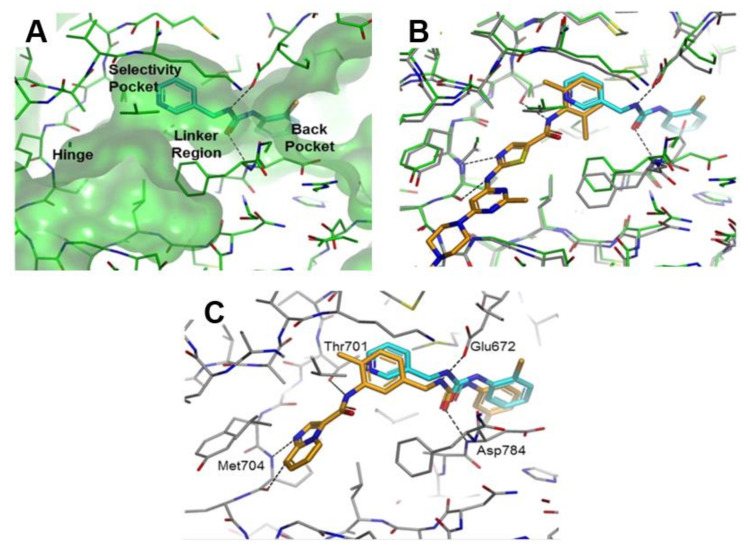
(**A**) Fragment 1 (**33**) in DDR1 binding pocket; (**B**) Cocomplex of dasatinib (**1**) and fragment 1 (**33**) (**C**) Cocomplex of compounds **33** and **34**. Reprinted with permission from Ref. [142].

Murray et al., in Astex Pharmaceuticals Co. screened ~1500 small molecules from a fragment library against DDR1 using a protein thermal shift assay. They discovered several compounds that were able to bind at the hinge or in the back pocket associated with the DFG-out conformation of DDR1, such as the multitargeted kinase inhibitor dasatinib (**1**). Compound **33** (Fragment 1, Figure 11) possessing a chlorophenyl moiety placed in the back region and a pyridyl group in the selectivity pocket proximal to the small gatekeeper residue (Thr701 in DDR1/2, Figure 12A), was selected as the starting fragment for the computational tool, AstexMerge. Accordingly, design A (Figure 11) was suggested by AstexMerge, based on the superimposition with dasatinib (Figure 12B). Further modification of design A by replacing the thiazole hinge binder of dasatinib with imidazopyridine led to design B (Figure 11). Using this fragment-based drug design, novel DDR1/2 inhibitors were generated [142].

All resulting compounds exhibited potent DDR2 inhibition activity in cells and displayed promising PK properties. The SAR study showed that the imidazopyridine motif improved DDR2 affinity and inclusion of methyl group in the selective pocket region in design B demonstrated a 30-fold increase in affinity. The best compound (**34**, Figure 11) showed an IC_50_ value of 3.3 nM towards DDR2 and an IC_50_ = 54% at 1.5 nM towards DDR1. Compound **34** inhibited a number of tyrosine kinases and was particularly effective against c-kit (IC_50_ = 19 nM) and significantly reduced basal and collagen I-stimulated DDR2 phosphorylation in both wild type DDR2- and mutant DDR2-expressing HEK293 cells. The binding mode of **34** superimposed on the starting fragment **33** inside DDR1 active site (Figure 12C) showed that the imidazopyridine was able to form essential hydrogen bonds with the hinge region.

**Figure 13:**
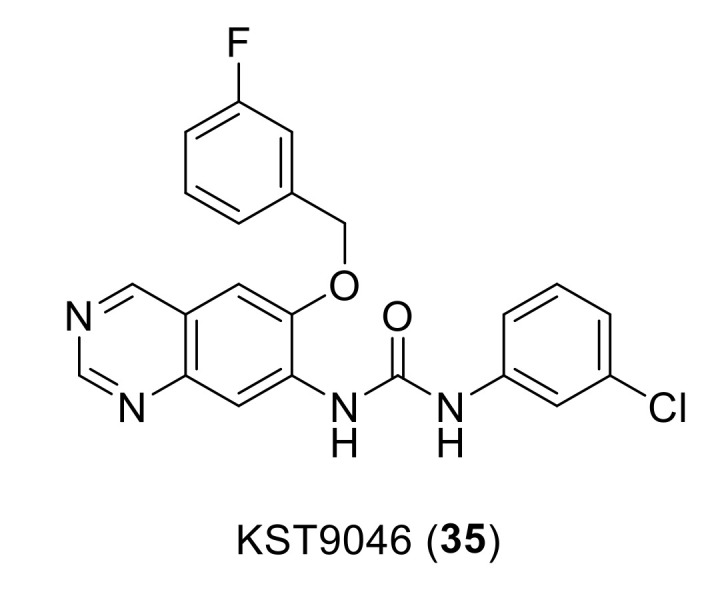
Chemical structure of compound KST9046 (**35**).

Compound KST9046 (**35**, Figure 13) is a recently reported DDR1 inhibitor with 6,7-disubstituted quinazoline-urea scaffold discovered by Elkamhawy et al., from Korea Institute of Science and Technology (KIST) [143]. Since 6,7-disubstitution of quinazoline ring is not common for kinase activity, a unique selectivity for DDR1 was found when the compound was checked over a large panel of 347 different kinases; the screening results showed a remarkable selective inhibitory activity over DDR1 kinase with IC_50_ of 4.38 µM. KST9046 was identified as a possible type III inhibitor for DDR1 kinase with an allosteric mode of interaction, which could explain its selectivity. A broad-spectrum activity of compound **35** with GI_50_ ranging from 1.3 to 3.9 µM over the 60 cell-line tumor panel of National Cancer Institute (NCI). Moreover, a promising low toxicity profile against four different isoforms of CYP450 was presented. Thus, compound **35** was reported as a promising lead for the development of broad-spectrum DDR1 selective antiproliferative candidates.

**Figure 14:**
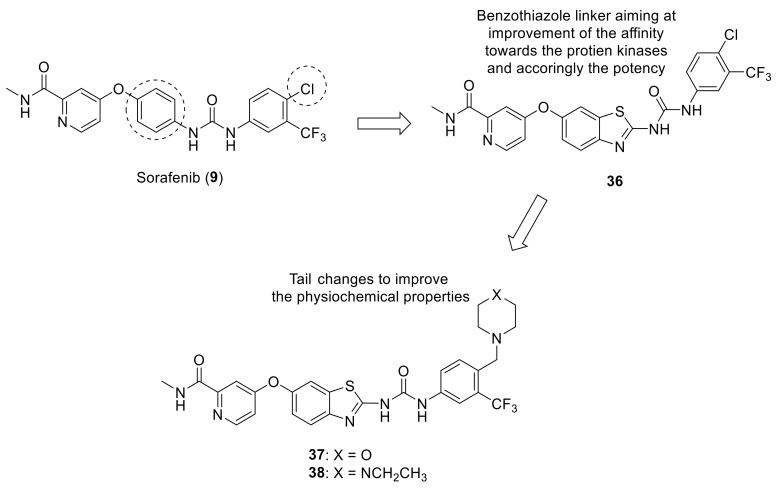
Structure-based design strategy of compounds **36**–**38** derived from sorafenib (**9**).

Recent studies from KIST by El-Damasy et al., attempted modifications to sorafenib (**9**) in order to increase its activity as a multi-target kinase inhibitor and derive more potent analogs. Using structure-based design strategy, the central phenyl linker of sorafenib was replaced with either quinoline moiety [144] or benzothiazole scaffold (**36**, Figure 14) [145] while maintaining the other structural features. This resulted in a remarkable increase in the cellular antiproliferative potency as well as favorable inhibitory activity toward B-Raf (V600E) and C-Raf kinases. However, the physiochemical properties needed further improvement, which was carried out through replacing the small lipophilic chlorine atom with either (morpholin-1-yl)methyl (**37**, Figure 14) or (4-ethylpiperazin-1-yl)methyl moieties (**38**, Figure 14) [146]. These two compounds were evaluated for their antitumor effects over a panel of 60 human cancer cell lines where they exhibited promising antiproliferative effects over numerous cell lines such as HL-60 (leukemia), HOP-92 (non-small cell lung cancer), HCT-116 (colon cancer), SF-5339 (CNS cancer) and SK-MEL-28 (melanoma). Both compounds were also subjected to further testing against a panel of 50 oncogenic kinases, where compound **38** potently inhibited 23 kinases including ABL-1, LYN, DDR1, and DDR2. Additionally, when compound **38** was tested against the human foreskin fibroblast (HFF-1) normal cell line, it demonstrated a relatively safe cytotoxicity profile (10.36 ± 0.19% inhibition). Based on these data, the promising inhibitory activity of compound **38** over DDR1 coupled with its safe cytotoxicity presented an ideal candidate for further development to enhance its selectivity against DDRs.

**Figure 15:**
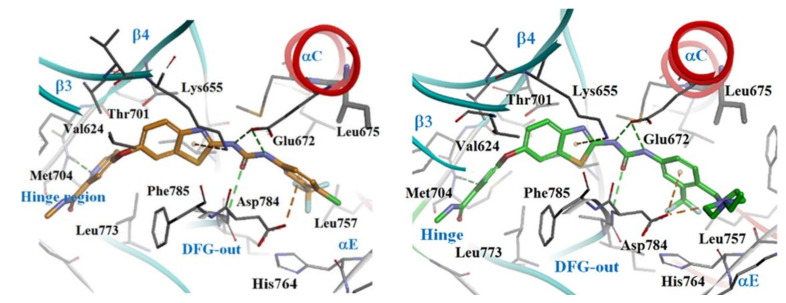
Docking models of compounds **36** (orange) and **38** (green) into DFG-out conformation of DDR1. Reprinted with permission from Ref. [146].

To understand the elicited kinase inhibitory activities of both compounds (**36** and **38**), a molecular docking study was carried out as demonstrated in Figure 15. The hydrogen bond formed by both compound **36** and **38** with the Met704 residue in the kinase hinge region via their pyridine nitrogen into the ATP binding site of DDR1 was found to be a large contributing factor to their activity, making the pyridine moiety essential for activity. Similarly, the urea moiety proved to be essential for activity through its ability to form three hydrogen bonds with Asp784 and Glu672. Additionally, the *m*-trifluoromethylphenyl group of **36** and **38** was predicted to be essential for activity as well through its ability to form hydrophobic interactions with the backbone Asp784 residue in the DFG region.

**Figure 16:**
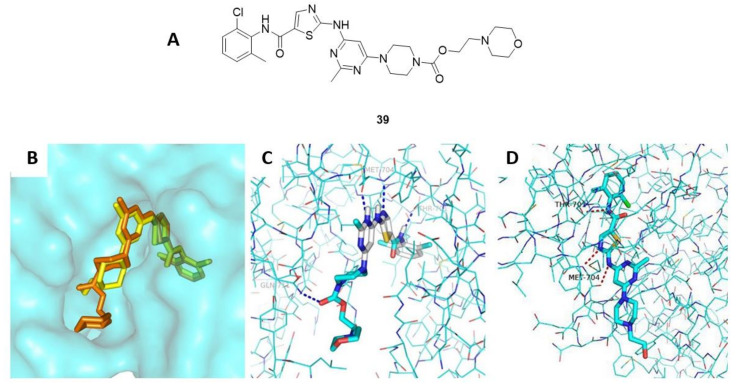
(**A**) Chemical structure of compound **39**; (**B**) Both compounds **39** (orange) and dasatinib (**1**) (yellow) overlayed in the active site residue of DDR1 kinase (PDB ID: 5BVW); (**C**) docking of compound **39** within DDR1 active site (**D**) Docking of dasatinib (1) within DDR1 active site. Reprinted with permission from Ref. [147].

In another study, Liu et al., synthesized novel dasatinib derivatives with potent DDR1 and DDR2 inhibitory activities [147]. One of the synthesized compounds, compound **39** (Figure 16A), compared with the parental dasatinib (**1**), demonstrated a considerably superior inhibitory potency over both DDRs and K562 cell lines (IC_50_ values of 2.26 ± 0.46 nM for DDR1, 7.04 ± 2.90 nM for DDR2, and 0.125 ± 0.017 nM for K562 cell line). Upon performing a comparative docking study for both the parental dasatinib (**1**) and compound **39** over DDR1 kinase (Figure 16B–D), they had almost the same estimated binding free energies of −9.7 and −9.6 kcal/mol, respectively. This suggested that the addition of the morpholine ring may have improved PK of the parent dasatinib compound leading to increased biological activity. Due to the potent activity of compound **39**, it could also be a promising therapeutic agent itself for additional assessments in a variety of disease models related to pathological DDR1 or DDR2 activities.

**Figure 17:**
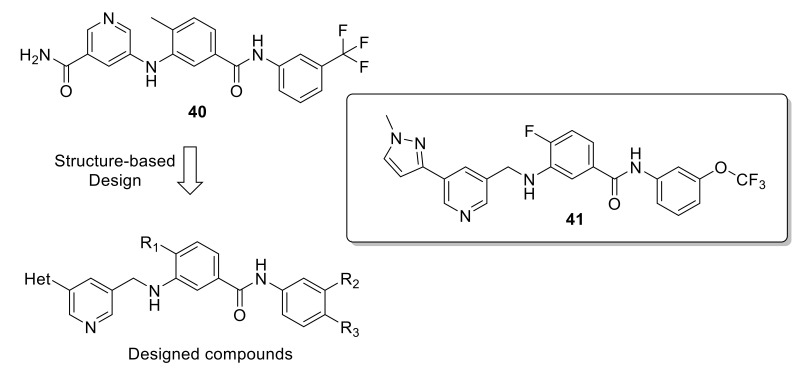
Structural modifications to compound **40** and chemical structure of compound **41**.

Jeffries et al., also attempted to synthesize a selective DDR1 inhibitor [148] through using compound **40** (Figure 17) as starting lead, which was initially identified as a selective DDR2 inhibitor (IC_50_ = 18.6 nM), although it inhibited DDR1 with an IC_50_ value of 12.4 nM [149]. Despite the high potency of compound **40** against DDR1, it demonstrated a poor drug metabolism and PK profile, as well as cytotoxicity at low concentrations. Therefore, Jeffries et al., modified its chemical structure as illustrated in Figure 17. However, these efforts failed to meet its original goal of synthesizing a selective DDR1 inhibitor. Instead, they produced compound **41** (Figure 17) which was proved to be a dual DDR1/2 inhibitor, with improved potency compared to compound **40** (DDR1 IC_50_ = 4.67 nM, DDR2 IC_50_ = 7.39 nM). This coupled with the fact that compound **41** showed enhanced binding and cell-based potency, augmented physiochemical and PK properties, make it an ideal candidate for further development.

**Figure 18:**
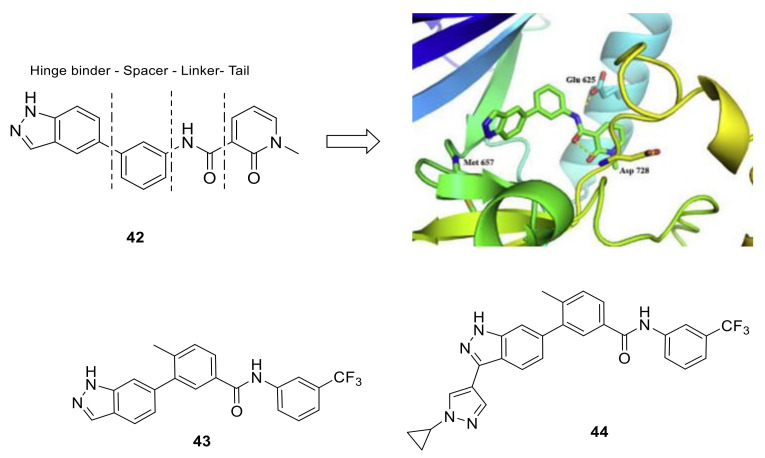
Chemical structures of compounds **42**–**44** and docking model of compound **42** into DDR2 active site. Reprinted with permission from Ref. [150].

Wang et al., discovered and optimized a novel series of 3-substituted indazole analogs as non-selective kinase inhibitors for lung squamous cell carcinoma [150]. Hit compound **42** (Figure 18), found by in-house library, showed moderate inhibitory activity in DDR2 inhibition assay. Through the docking prediction, compound **42** combined with hinge segment of DDR2 as a typical Type-II DFG-out kinase inhibitor (Figure 18) where nitrogen atoms in the indazole ring and the hinge segment of DDR2 formed essential hydrogen bonds. In addition, the interaction with nearby residues Glu625 and Asp728 has been enhanced by the amide group. When SAR optimization was carried out over the four parts of compound **42** (Hinge binder, Spacer, Linker and Cap part, Figure 18), compound **43** (Figure 18) was obtained with an IC_50_ value of 1.5 ± 0.1 nM against DDR2. A further optimization afforded four compounds with enhanced biological activity, among them, compound **44** (Figure 18) exhibited remarkable PK properties with a good exposure and moderate half-life of 3.05 h. In addition, **44** also showed a certain selectivity in in vitro enzymatic screen assays. Compound **44** also inhibited DDR2 phosphorylation and suppressed tumor growth with tumor growth inhibition rates (TGI) of 82.8% in mice bearing NCI-H2286 (DDR-dependent cell line) at doses of 10 mg/kg for 10 consecutive days. According to this study, compound **44** may encourage further research on drug development of lung squamous cell carcinoma.

**Figure 19:**
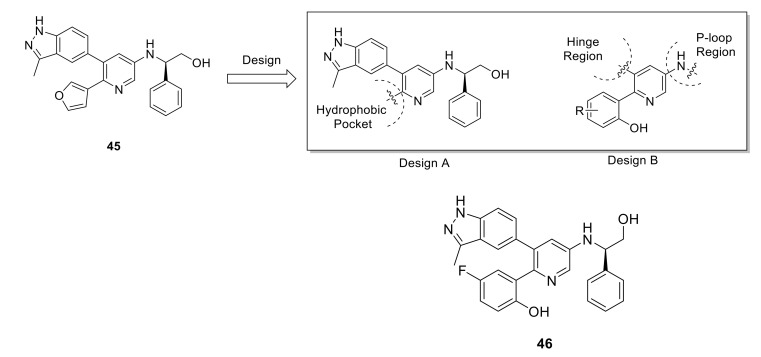
Structural-based design and chemical structures of compounds **45** and **46**. Reprinted with permission from Ref. [151].

Zhu et al., reported a novel series of pyridin-3-amine derivatives as protein kinase inhibitors for NSCLC treatment [151]. As demonstrated in Figure 19, hit optimization of compound **45** led to SAR exploration of the new pyridine-3-amine derivatives. Among all synthesized compounds, compound **46** demonstrated nanomolar IC_50_ values for several NSCLC-related oncogene kinases including FGFR1–3 (18.0, 1.6, and 27.5 nM, respectively), RET (0.2 nM), KDR (1.7 nM), EGFR/T790M/L858R (6.0 nM), DDR2 (0.8 nM), and ALK 101.1 nM). In cell assay over SNU16 cell line, it exhibited an IC_50_ value of 24.8 nM. Furthermore, compound **46** demonstrated a strong antitumor efficacy (TGI = 66.1%) in NCI-H1581 NSCLC xenografts with a favorable PK profile in vivo. Despite the low selectivity ratio of compound **46**, its high potency against DDR2 kinase and its good PK properties are two main factors that could be taken into consideration for further development of novel DDR2 inhibitors based on the pyridin-3-amine scaffold.

**Figure 20:**
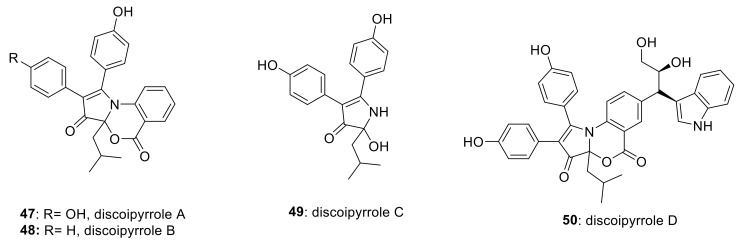
Chemical structures of natural alkaloids discoipyrroles A–D (**47**–**50**). Reprinted with permission from Ref. [153].

Using a functional signature-based ontology map approach to identify the signaling pathway/molecular target of natural products, Hu et al., discovered a family of alkaloid natural products (discoipyrroles A–D, **47**–**50**, Figure 20) from *Bacillus hunanensis* possessing strong inhibitory activity against DDR2-dependent migration of BR5 fibroblasts [152]. Discoipyrroles A–D exhibited potent and selective cytotoxicity toward DDR2 mutant lung cancer cell lines with a range of IC_50_ values of 120–400 nM. Three years later, due to their potent activity against DDR2, Zhang et al., attempted various methods to synthesize these naturally occurring compounds [153].

**Figure 21:**
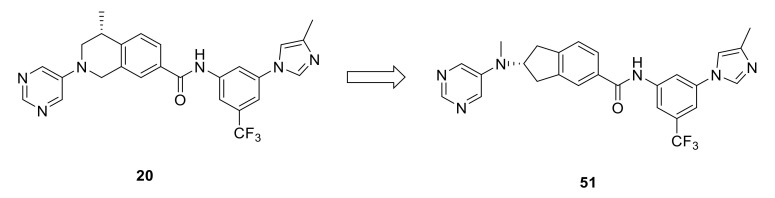
Structural-based design of compound **20** to afford compound **51**. Reprinted from Ref. [154].

Recently, while Zhu et al., discovered a new series of 2-amino-2,3-dihydro-1*H*-indene-5-carboxamides as selective DDR1 inhibitors using a structure-based design strategy based on compound **20** [154], their efforts generated compound **51** (Figure 21) which showed a unique ability to bind to the ATP-binding site of DDR1 with a *K*_d_ value of 5.9 nM and an IC_50_ value of 14.9 nM. In addition, compared to **20**, compound **51** had slightly lower potencies against all off-target kinases and effectively inhibited collagen-induced activation of DDR1 as well as its downstream signaling proteins. Compound **51** also inhibited DDR1-mediated cadherin switching, suppressed colony development of pancreatic cancer cells, exhibited good PK properties, and displayed a promising therapeutic activity through oral administration in orthotropic syngeneic pancreatic cancer models.

**Figure 22:**
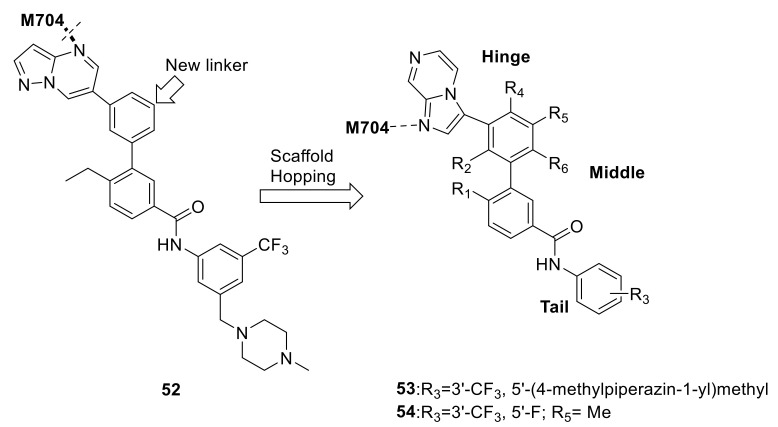
Structural-based design of compound **52**–**54**. Reprinted with permission from Ref. [155].

Mo et al., also recently designed a new carboxamide series as selective DDR1 inhibitors via incorporating a novel substituted phenyl linker in place of the alkyne group in compound 7RH (**18**) [155]. Although the preliminary design led to a higher selectivity for DDR1 kinase by compound **52** (Figure 22), there was a 50-fold loss of activity against DDR1. Modeling studies suggested that the reason for the activity loss was that compound **52** was not able to fit tightly into the DDR1 ATP binding pocket and could not achieve the essential hydrogen bond with Met704. Thus, imidazo[1,2-*a*]pyrazine moiety was used to replace the pyrazolo[3,4-*b*]pyridine to restore the key hydrogen bond with Met704. Further optimizations led to compound **53** (Figure 22) which exhibited excellent inhibitory activity against DDR1 with an IC_50_ value of 9.6 nM, but its selectivity against other homologous kinases was decreased. Modeling simulations showed that compound **53** bound to DDR1 with a classical type II binding mode. The best compound (**54**, Figure 22) displayed potent inhibitory activity against DDR1 with an IC_50_ value of 23.8 nM with an extraordinary target selectivity. Moreover, the in vivo study revealed that compound **54** suppressed colony formation, migration, and invasion of H1299 NSCLC.

**Figure 23:**
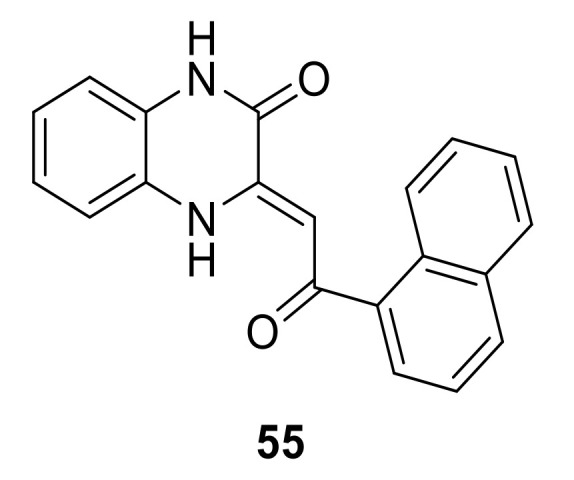
Chemical structure of compound **55**.

During the research for development of c-Jun N-terminal kinase 3 (JNK3) new inhibitors, Dou et al., discovered that a potent JNK3 inhibitor (**55**, Figure 23) possessing a 3,4-dihydroquinoxalin-2(1*H*)-one core structure displayed an interesting DDR1 inhibition activity (IC_50_ = 0.16 µM) [156]. The modeling study of compound **55** into the active site of DDR1 indicated that the oxygen atom in the quinoxaline core and hydrogen atom of NH formed two hydrogen bonds to the NH and backbone carbonyl of Met704. Thus, further optimization of compound **55** may lead to the development of more potent and selective quinoxaline-based DDR1 inhibitors.

**Figure 24:**
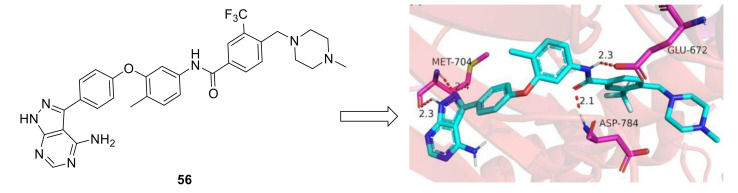
Chemical structure of compound **56** and its docking model to DDR1 active site. Reprinted with permission from Ref. [157].

Lately, Dong et al., reported the synthesis and evaluation of new 4-amino-1*H*-pyrazolo[3,4-d]pyrimidin derivatives as DDR1 inhibitors [157]. Compound **56** (Figure 24) which showed the most potent DDR1 inhibitory activity (IC_50_ value = 0.044 µM) exhibited potent inhibition against cell proliferation in HCT-116 and MDA-MB-231 cell lines (IC_50_ = 4.00 and 3.36 μM respectively). The conducted modeling studies (Figure 24) revealed that it was able to bind into DDR1 kinase active site via four hydrogen bonds, two π-π stacked interactions, electrostatic interactions, and various Van der Waals interactions.

**Figure 25:**
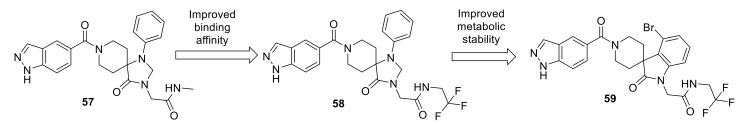
Chemical structure of compounds **57**–**59**.

Another series of potent and selective DDR1 inhibitors were synthesized and reported by Richter et al. They employed a parallel DNA encoded library screening against DDR1 and DDR2 to uncover a series of small molecules which exhibited a potent selectivity for DDR1 over DDR2. Figure 25 depicts the structural optimization process utilized to reveal compound **59** (DDR1 IC_50_ = 0.029 µM and DDR2 IC_50_ = 1.9 µM) starting from the lead compound **57** (DDR1 IC_50_ = 1.4 µM and DDR2 IC_50_ > 167 µM) followed by compound **58** (DDR1 IC_50_ = 0.016 µM and DDR2 IC_50_ = 6.3 µM). Compound **59** possessed a remarkable kinome selectivity coupled with promising in vitro safety profile, PK, and physicochemical properties. Richter et al., through a series of structural modifications not only managed to enhance the potency and selectivity of the final compound **59** but also increase the metabolic stability of the synthesized compound, with a clearance rate of 10 µL/min/mg in both mouse and human [158].

## 7. Conclusions

Throughout this review, we discussed the biological roles DDRs, their relation to different human diseases, mainly for cancer, and the medicinal chemistry approaches used in the journey of development of DDR1 and DDR2 kinase inhibitors since the discovery of these targets. Since great efforts have been made in this area of research, a number of selective and potent DDR1 and DDR2 inhibitors have been reported and discussed in this review. Furthermore, the available molecular docking studies allowed us to predict that a potent DDR1 inhibitor must contain certain structural features. One such essential structural feature is an amide linker that facilitates the formation of a hydrogen bond with GLU672 and an additional hydrogen bond with the backbone NH of ASP784. A moiety able to form hydrogen bonding with the “gate keeper” amino acid THR071 is another essential feature a potent and selective DDR1 inhibitor must possess. The ability of the synthesized compound to form a hydrogen bonding with MET704 as well as containing a hydrophobic moiety in its “tail” are two structural features that are predicted to further increase the binding affinity of any synthesized molecule with the DDR1 active site residue.

In addition to the classical methods of drug design, many researchers are currently attempting to apply new strategies to develop DDR inhibitors; for instance, ligand-based pharmacophore mapping [159], integrative transcriptome meta-analysis [160], and deep learning [161,162,163,164]. However, given the fact that the pathological roles of DDR1 and DDR2 are not yet fully understood, further chemical biology research is highly needed to gain further understanding of these vital kinase targets. As discussed, several potent small molecule inhibitors were found in literature, for example, compounds **34**, **39**, and **40** (IC_50_ < 5 nM), however, major concerns related to selectivity, PK properties, mutation resistance, and safety of the promising inhibitors needs to be addressed. Since no selective DDR1 or DDR2 inhibitor has been moved into clinical investigation to date, we believe that further medicinal chemistry research aiming at SAR improvement of the most promising compounds highlighted in this review as well as design of DDR1 and DDR2 allosteric modulators targeting pockets out of DDR conserved kinase domain may play a significant role to develop more potent, selective, and safe inhibitors. Furthermore, since many of the discussed small molecule DDR inhibitors were found to share the same “skeleton”, a 3D QSAR study utilizing their known IC_50_ values would be key to the synthesis of new structurally modified DDR inhibitors with higher selectivity and potency.
